# Impaired *CPT1A* Gene Expression Response to Retinoic Acid Treatment in Human PBMC as Predictor of Metabolic Risk

**DOI:** 10.3390/nu12082269

**Published:** 2020-07-29

**Authors:** Margalida Cifre, Andreu Palou, Paula Oliver

**Affiliations:** 1 Nutrigenomics and Obesity Group, University of the Balearic Islands, 07122 Palma, Spain; marga.cifrec@gmail.com (M.C.); paula.oliver@uib.es (P.O.); 2 CIBER of Pathophysiology of Obesity and Nutrition (CIBEROBN), 28029 Madrid, Spain; 3 Health Research Institute of the Balearic Islands (IdISBa), 07010 Palma, Spain

**Keywords:** blood cells, biomarkers, retinoic acid, obesity, HDL-cholesterol

## Abstract

Ex vivo human peripheral blood mononuclear cell (PBMC) systems offer the possibility to test transcriptomic effects of food bioactive compounds with potential health effects. We investigated all-trans retinoic acid (ATRA) effect on mRNA expression of key lipid metabolism and inflammatory genes in PBMCs from normal-weight (NW) and overweight-obese (OW-OB) men with different metabolic syndrome-related features. PBMCs were incubated with 10 µM ATRA and mRNA levels of selected genes were analyzed using real-time RT-qPCR. Human ex vivo PBMCs responded to ATRA treatment, but the response for some genes was dependent on body mass index (BMI), with a lower response in PBMC from OW-OB than from NW donors. Moreover, gene expression response was affected by circulating high-density lipoprotein (HDL)-cholesterol levels. Particularly, the response to ATRA of *CPT1A*, previously reported as a sensitive metabolic risk predictive biomarker, was dependent on HDL levels and not on BMI, being impaired in those individuals with lower HDL levels, specifically in OW-OB. Thus, PBMCs’ insensitivity to ATRA, which can be considered as indicative of impaired metabolism, was observed in individuals with higher metabolic risk (OW-OB with low HDL levels). In conclusion, an ex vivo human PBMC system indicates that ATRA response could be influenced by metabolic syndrome features. Moreover, our study reinforces the role of *CPT1A* as a marker of metabolic risk and points to plasmatic HDL-cholesterol levels as a parameter to take into consideration when the effects of nutritional factors and/or dietary interventions on humans are under study. Further studies including women are required to detect potential gender differences in the observed effects.

## 1. Introduction

In recent years, metabolic-related health problems have emerged as a result of the increasing incidence of obesity and metabolic alterations linked to metabolic syndrome [1]. Both obesity and metabolic syndrome play a crucial role in the origin of type 2 diabetes and cardiometabolic diseases [2]. Nowadays, the available treatments against these metabolic abnormalities are poorly effective; therefore, prevention strategies are those that should receive more attention. In this sense, peripheral blood mononuclear cells (PBMCs), a subset of blood cells consisting of lymphocytes and monocytes, are being consolidated as a biological tool for the search of health metabolic biomarkers as they are easy to obtain and their gene expression is representative of whole metabolic status [3,4]. Our group previously reported that PBMCs could reflect the response to nutritional interventions, metabolic alterations or other pathophysiological conditions related to obesity at level of gene expression both in human and animal models [5,6,7,8,9,10]. Moreover, we have shown that human PBMCs maintained ex vivo represent an interesting tool to test the effects of food bioactive compounds and to discern the differential transcriptomic response to those compounds related to increased body weight [11].

Previous studies from our group showed that retinoic acid (RA), the active metabolite of vitamin A, reduces body weight and adiposity [12,13,14] and ameliorates glucose tolerance, insulin sensitivity, dyslipidemia, and hepatic steatosis in diet-induced obese mice [15,16,17]. It is known that a large part of these beneficial effects is mediated through the action of RA on adipose tissue, a key tissue whose expansion has been related to the development of obesity and other metabolic disorders. The role of RA regarding metabolism of adipose tissue has been generally proven in vitro. It has been demonstrated that RA is involved in the control of adiposity, affects adaptive thermogenesis, and promotes increased fatty acid oxidative metabolism [18,19]. However, apart from animal studies, evidences suggesting a potential role of RA in the control of adiposity and other factors related to metabolic abnormalities in human populations are very limited. In fact, contradictory data appear on the effect of vitamin A and its derivatives on humans [20]. However, although evidences exist pointing to a connection of vitamin A or RA and metabolic risk, human studies do not provide consistent associations with obesity or insulin resistance [20,21]. Moreover, the majority of preclinical and clinical studies agree that an adequate vitamin A/RA status has a preventive role on cardiovascular health, while an excess or deficiency can increase the cardiovascular risk [22]. Interestingly, although only a few studies exist on RA, it has been shown to be inversely correlated with metabolic syndrome and cardiovascular risk [20,23]. Thus, there is a need of additional research to clarify RA’s role on health, and its mechanisms of action. The possibility to have an ex vivo system of human PBMCs whose gene expression are representative of whole metabolic status would be extremely useful to test the effects of RA without the need of administering it (avoiding potential problems of toxicity). It is well known that RA exerts its biological effects in large part by controlling gene expression. RA acts through the binding and activation of members of the nuclear receptor family including retinoic acid receptors (RARs) and retinoid X receptors (RXRs), both existing in different isoforms [18]. In addition, RXRs serve as obligate heterodimeric partners for other nuclear receptors, including peroxisome proliferator-activated receptors (PPARs) deeply involved in the control of lipid metabolism [18]. The activation of these receptors elicits a modulation of key inflammation and metabolic pathways related to lipid metabolism, two processes linked to the development of obesity and metabolic-related abnormalities. Both retinoid receptors and PPARs are expressed in PBMCs [24,25].

Considering the usefulness of PBMCs to obtain biomarkers related to nutrition and obesity [4], here we investigated the action of all-trans retinoic acid (ATRA) on the expression of key inflammatory and lipid metabolism genes on an ex vivo system of PBMCs from normal-weight and overweight-obese humans and its relation with metabolic syndrome-related features. Our first aim was to determine the usefulness of this surrogate system to study ATRA effect analyzing potential differential outcomes depending on body weight. When analyzing the data, we realized that the effect of ATRA on gene expression was not dependent only on body mass index but on the circulating high-density lipoprotein (HDL)-cholesterol levels. Results obtained for *CPT1A* expression, previously reported as a biomarker of diet-related metabolic alterations [7,26], appeared of particular interest. Thus, subsequently, additional subgroups were made based on circulating HDL levels of the PBMC donors and of the degree of *CPT1A* response to ATRA treatment, to find out biomarkers of metabolic risk beyond the typical ones in order to provide more personalized diagnostic tools.

## 2. Materials and Methods

### 2.1. Subjects 

Experiments were performed with blood samples collected from volunteers of the NUTRI-BLOOD study. Informed consent to participate in the study was obtained from all volunteers. The study was conducted in accordance with the Declaration of Helsinki, and the protocol was approved by the Ethics Committee of Research of the Balearic Islands (CEI-IB) (NUTRI-BLOOD study, no. IB 2114/13 PI). Eighteen apparently healthy men aged 19–36 years were divided into two groups according to their body mass index (BMI): normal-weight (NW) group (BMI <25 kg/m^2^) and overweight-obese (OW-OB) group (BMI ≥25 kg/m^2^) composed of 9 subjects each. The number of individuals per group was selected based on what is usual in previous ex vivo experiments performed with human PBMCs focused on gene expression analysis (e.g., [27,28]), as well as on our own preliminary observations. NW group was considered as the control group. The inclusion criteria were as follows: men aged 18–45 years not suffering from chronic disease, not taking regular medication or drugs, keeping their habitual diet, and non-smokers. The study was performed only with males to avoid any potential interference, which could appear due to menstrual cycle hormonal fluctuations occurring in women. Blood was obtained after nocturnal fast. The same day of blood collection, anthropometric (weight, height, BMI, kg of fat, % of fat and blood pressure) and blood parameters (glucose and triglycerides) were also collected.

Anthropometric and most relevant circulating parameters of the volunteers were previously reported [11]. In brief, compared to NW group, OW-OB subjects displayed increased body weight, BMI, fat content, and higher blood pressure (both systolic and diastolic). OW-OB group showed glucose, triglycerides, total and low-density lipoprotein (LDL)-cholesterol levels comparable to those in the NW group. However, they presented signs of insulin resistance, such as increased fasting circulating insulin levels and a trend for increased homeostatic model assessment for insulin resistance (HOMA-IR) index (*p* < 0.1), as well as lower plasma HDL-cholesterol. Moreover, the proportion of lymphocytes and monocytes in PBMC samples was not different between both groups (an average of 84% lymphocytes and 16% monocytes), discarding in this way that differences in gene expression when analyzing the PBMC samples could be influenced by differences in the amount of the two cell types. During the process of data analysis, subjects were divided into additional groups/subgroups. On one hand, all the subjects participating in the study were separated into two groups considering their circulating HDL levels (low or high). The low HDL group was composed of 6 individuals with HDL levels <40 mg/dL, using as cut-off valuethat established in the National Cholesterol Treatment Panel guidelines [29]. The high HDL group was composed of 12 individuals with HDL levels >40 mg/dL. Moreover, subjects of the NW and OW-OB groups were divided into two subgroups considering the degree of *CPT1A* gene expression response of their PBMCs treated ex vivo with ATRA (i.e., high or low *CPT1A* response). Median *CPT1A* response to ATRA in ex vivo PBMCs from all donors (with independence of their range of body weight) was established as the split point. The use of median as separating threshold value to dichotomize subjects into high vs. low responders is commonly used in basic and clinical research. A high response was considered when *CPT1A* expression increased 16% over the baseline value. In this way, four different subgroups were obtained: NW with high (*n* = 4) or low (*n* = 5) *CPT1A* response and OW-OB with high (*n* = 5) or low (*n* = 4) *CPT1A* response. The sample size, even after subgrouping, was appropriated for ex vivo experiments in which the conditions are more controlled than in intervention studies.

### 2.2. All-Trans Retinoic Acid Preparation

ATRA was purchased from Sigma-Aldrich (St. Louis, MO, USA) and was prepared dissolved in absolute ethanol as a stock solution (10 mM), stored in single use aliquots at −80 °C to prevent repeated freeze thaw cycles and protected from light. A final concentration of 10 µM ATRA was administered to human PBMCs maintained ex vivo. This concentration has been proven non-toxic in other studies using different cell types, including PBMCs [30]. We tested the 10 μM dose on PBMCs from a pool of two overweight-obese individuals to determine % of cytotoxicity of ATRA treatment based on the measurement of LDH activity released from damaged cells (Roche Diagnostics, Barcelona, Spain). The toxicity test was performed twice with samples coming from triplicate cultures of ATRA-treated and non-treated cells. In one of the tests, we observed up to a maximum of 15% cytotoxicity (lysis) in 10 µM ATRA-treated cells. However, the average results from both tests showed no difference in the percentage of cytotoxicity: 0% for control and 0.7% for ATRA-treated cells. The maximum LDH release (i.e., positive control (100% of toxicity)) was obtained by adding a lysis reagent to control cells.

### 2.3. Isolation and Ex Vivo treatment of PBMCs

Venous blood from the volunteers was collected in EDTA tubes and diluted 1:1 with phosphate-buffered saline (PBS) 1×. PBMC isolation was performed by density gradient over Ficoll-Paque Plus (GE Healthcare Bio Science, Barcelona, Spain), with 30 min centrifugation at 400× *g*, 25 °C, with no break. After centrifugation, plasma samples were obtained (top layer) and stored at −80 °C. The PBMC layer was then collected and washed with PBS 1× (twice) by centrifugation for 10 min at 300× *g* (25 °C). Then, the obtained PBMCs were adjusted to 4 × 10^6^ cells/mL using sterile RPMI-1640 culture medium (Sigma-Aldrich, St Louis, MO, USA) supplemented with 10% fetal bovine serum, 1% L-glutamine (2 mM), 100 unit/mL penicillin, and 100 µg/mL streptomycin (reagents from Sigma-Aldrich, St. Louis, MO, USA). A final concentration of 1 × 10^6^ cells from NW and OW-OB donors was maintained in suspension in 24-well plates. Cells were activated using CD3/CD28 magnetic beads (one bead each two cells) (Life Technologies, Madrid, Spain). Each well was treated with ATRA diluted in RPMI-1640 culture medium in a final concentration of 10 µM. The original ATRA solution stock was dissolved in ethanol that was present in a final concentration of 0.1% in the treated wells; an equivalent dose of ethanol was added to control cells. Additionally, PBMC cultures from three non-obese participants were set up to evaluate the dose-dependent effects of ATRA; hence, 1 and 10 µM of this compound were assessed. The dose-dependent test was performed directly in normal-weight subjects as we previously reported the insensitivity of ex vivo PBMCs to food bioactives as a result of increased body weight [11]. The cultures were maintained at 37 °C in an atmosphere of 5% CO_2_ for 48 h. The 48 h incubation period was chosen based on our previous optimization experiments, which showed a maximum effect on gene expression modulation in comparison to a 24 h incubation period, without affecting cell viability.

### 2.4. RNA Extraction and Real-Time Reverse Transcriptase Polymerase Chain Reaction (RT-qPCR)

Total RNA was isolated from human PBMC after 48 h ex vivo incubation with ATRA, using Direct-zol™ RNA Mini-Prep (Zymo Research Corp, Irvine, CA, USA). RNA integrity was confirmed by agarose gel electrophoresis. RNA (0.05 µg) was transcribed into cDNA using an iScript™ cDNA synthesis kit (Bio-Rad Laboratories, Madrid, Spain) in an Applied Biosystems 2720 Thermal Cycler. For each PBMC sample, three replicates were reverse-transcribed, and real-time qPCR was performed for each RT product (three PCRs per sample). The mRNA expression of inflammatory and energy metabolism genes of interest was analyzed. Each PCR was carried out as previously described [31]. To calculate the threshold cycle (Ct) the instrument software (StepOne Software v2.0, from Applied Biosystems) was used. The relative mRNA expression was calculated for each sample as a percentage referred to gene expression of control PBMC from NW group (which was set as 100%), using Livak’s method [32]. For data normalization the housekeeping gene ribosomal protein, large, P0 (*RPLP0*)/human acidic ribosomal protein (HuPO), which was selected from a panel of tested genes due to its higher stability among groups, was used. This gene has been previously described as a stable reference gene for isolated human PBMCs [33,34]. Primers were obtained from Sigma Genosys (Sigma Aldrich Química SA, Madrid, Spain), and are shown in Table 1.

### 2.5. Measurement of Produced Cytokines

Interleukin 6 (IL6) and tumor necrosis factor alpha (TNFα) concentrations were analyzed in the supernatants of the cell culture wells using specific sandwich ELISAs (RayBiotech, Norcross, GA, USA) to test cytokine released by PBMCs maintained ex vivo. Measurements were performed using the following dilutions: 1/50 for IL6, and 1/8 and 1/10 (culture medium from NW and OW-OB donors, respectively) for TNFα analysis.

### 2.6. Statistical Analysis

SPSS for windows (SPSS, Chicago, IL, USA) was used to perform statistical analysis. Shapiro–Wilk test and Levene test were used to test normal distribution of the data and homogeneity of variances, respectively. For statistical comparison of anthropometric and serum parameters between NW and OW-OB subjects, unpaired *t*-test was used (between-group analysis). Paired *t*-test was applied to compare gene expression changes between ATRA-stimulated PBMCs and baseline, in NW or OW-OB groups (within-group analysis). The same statistical analysis was applied to cytokine released to culture medium. Two-way repeated measures ANOVA was used to compare differences of ATRA treatment (pre- and post-treatment) of ex vivo PBMCs on gene expression or circulating cytokines depending on body weight. Two-way ANOVA was applied to determine differences between normal-weight and overweight-obese subjects and *CPT1A* response to ATRA regarding the cholesterol levels. Pearson correlations were performed to identify associations among *CPT1* response and HDL-cholesterol levels. One-way repeated measures ANOVA followed by least significant difference (LSD) post-hoc test was applied to evaluate the dose effects of ATRA on gene expression. If variables were not adjusted to parametric criteria, they were log transformed to perform statistical measures. Threshold of significance was defined at *p* < 0.05 for all analyses. Detailed explanation of the statistical analysis used is present in the figure/table legends.

## 3. Results

### 3.1. Dose-Response Effects of ATRA on Gene Expression of Isolated Human PBMsC Maintained Ex Vivo

**Figure 1:**
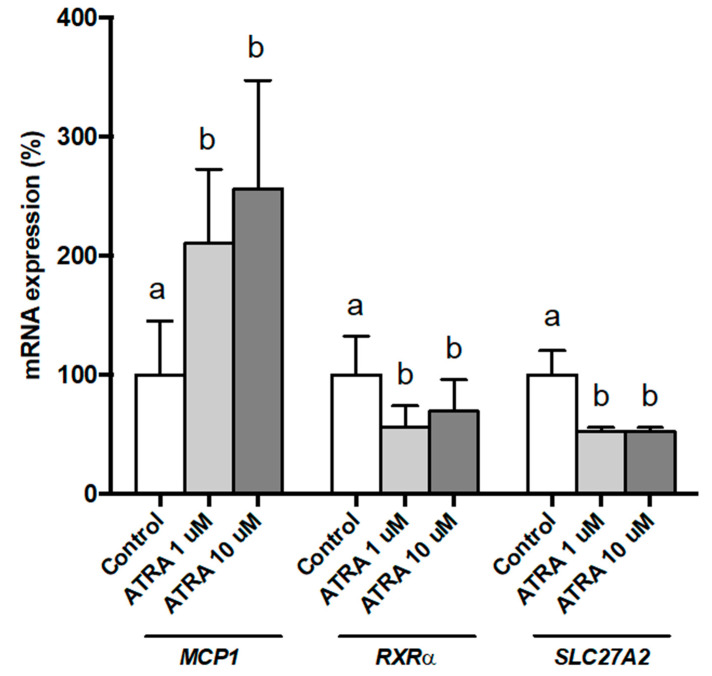
Dose effects of all-trans retinoic acid (ATRA) on *MCP1*, *RXRα,* and *SLC27A2* expression in human PBMCs from selected normal-weight (NW) individuals, measured by real-time RT-qPCR. PBMCs were treated for 48 h with vehicle control (ethanol) or ATRA at various concentrations in the presence of CD3/CD28 magnetic beads. mRNA levels were normalized to *RPLP0* and expressed relative to vehicle-treated cells. Data represent means ± SEM (3 replicates). One-way repeated measures ANOVA was performed followed by least significant difference (LSD) post-hoc test. Data not sharing a common letter (a,b) are significantly different (*p* < 0.05).

We wanted to test whether effects of ATRA treatment were dose-dependent. To this purpose, we studied this dose-response effect in PBMCs from three NW participants and analyzed the gene expression of *MCP1*, *RXRα,* and *SLC27A2* as selected genes (Figure 1). Results show that the effect of ATRA treatment on PBMC gene expression was significant for both doses used (1 and 10 µM). The effect of ATRA tended to be dose-dependent on *MCP1* expression but statistical significance between doses was not reached due to the large inter-individual variability in gene expression response. Concerning *RXRα* and *SLC27A2* expression, the effects of ATRA were similar with 1 and 10 µM.

### 3.2. Gene Expression of RXRα and RARα in Isolated Human PBMCs Treated with ATRA

**Figure 2:**
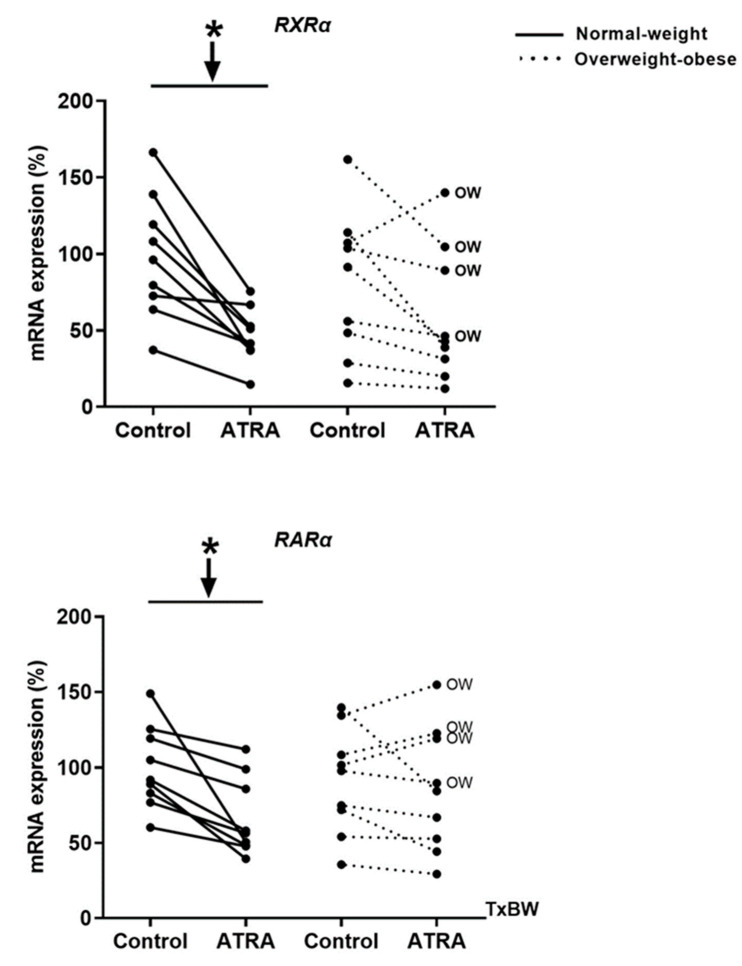
Effect of ATRA treatment on the mRNA levels of retinoid receptors (*RXRα* and *RARα*) in isolated PBMCs from NW and overweight-obese (OW-OB) individuals, measured by real-time RT-qPCR. Isolated PBMCs were treated for 48 h with a vehicle control (ethanol) or 10 μM ATRA in the presence of CD3/CD28 magnetic beads. All data are presented as individual responses and represent means of 3 replicates per subject and condition. mRNA levels were normalized to *RPLP0* and expressed relative to vehicle-treated cells. * *p* < 0.05 was calculated using a paired *t*-test. Two-way repeated measures ANOVA was performed to compare the different effect of treatments and body weight on gene expression. T × BW, interactive effect between treatment and body weight of PBMC donors (detailed results are shown in Table 2A). OW indicates the overweight subjects within individuals of the overweight-obese group. Down arrows represent decreased expression.

As shown in Figure 2, incubation of human PBMCs with 10 µM of ATRA decreased mRNA levels of both receptors *RXRα* and *RARα* in NW group as previously reported by Bonet et al. [35] in cultured brown adipocytes, as an auto-regulatory mechanism of the retinoids effects. However, *RXRα* and *RARα* expression remained unchanged in the OW-OB group.

### 3.3. Gene Expression and Release of Inflammatory Markers in Isolated Human PBMCs Treated with ATRA

**Figure 3:**
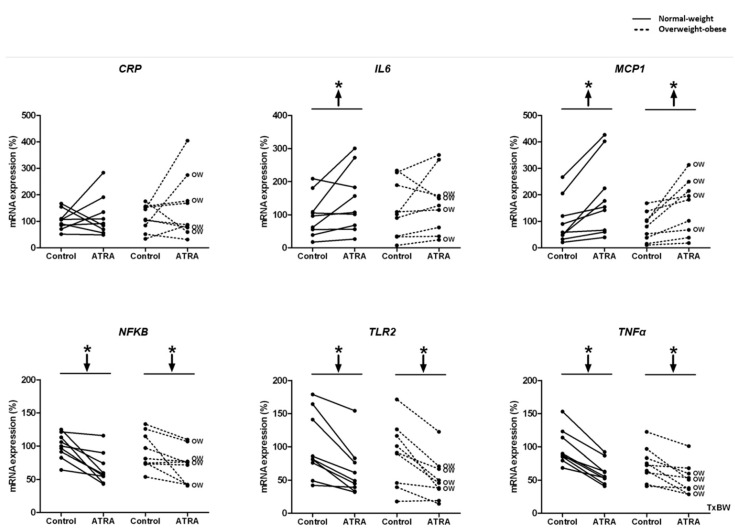
Effect of ATRA treatment on mRNA expression of gene expression of the inflammatory genes: *CRP*, *IL6*, *MCP1*, *NFKB*, *TLR2,* and *TNFα* in isolated PBMCs from NW and OW-OB individuals, in the same conditions described in Figure 2. * *p* < 0.05 was calculated using a paired *t*-test. Two-way repeated measures ANOVA was performed to compare the different effect of treatments and body weight on gene expression. T × BW, interactive effect between treatment and body weight of PBMC donors; if not indicated no interactive effect was found (detailed results are shown in Table 2A). OW indicates the overweight subjects within individuals of the overweight-obese group. Down arrows represent decreased expression while up arrows represent increased expression.

**Figure 4:**
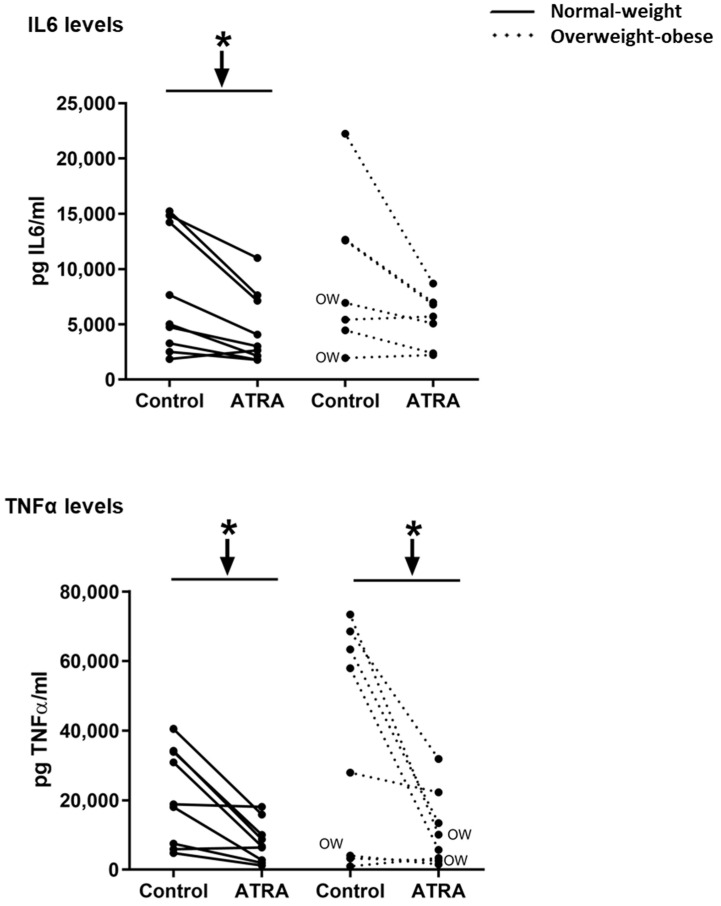
Effect of ATRA (10 μM) treatment on isolated PBMCs on IL6 and TNF*α* levels in the culture media. In the overweight-obese group, one culture media sample was missing, and thus only 8 out of 9 samples were measured. Additionally, also in this group, one outlier data was discarded for IL6 levels. Cytokine concentrations were detected by ELISA. All data are presented as individual responses and represent means of 2 duplicates per subject. * *p* < 0.05 was calculated using a paired *t*-test. Two-way repeated measures ANOVA was performed to compare the different effect of treatments and body weight on gene expression. No interactive effect between treatment and body weight of PBMC donors was found (detailed results are shown in Table 2B). OW indicates the overweight subjects within individuals of the overweight-obese group. Down arrows represent decreased expression.

Decreased mRNA levels of *NFΚB*, *TLR2,* and *TNFα* were observed in NW and OW-OB groups (Figure 3). On the contrary, the exposure to ATRA increased the transcriptomic levels of *MCP1* in PBMCs from both groups of participants and of *IL6* in NW group (Figure 3). However, the increased mRNA levels of *IL6* in this group were not reflected into higher levels of this cytokine in the culture media (Figure 4). In fact, the release of IL6 and TNFα in the culture media was lower in NW subjects, as well as TNFα release in OW-OB subjects, in response to ATRA treatment (Figure 4).

### 3.4. Gene Expression of Key Lipid Metabolism Genes in Isolated Human PBMC Treated with ATRA

**Figure 5:**
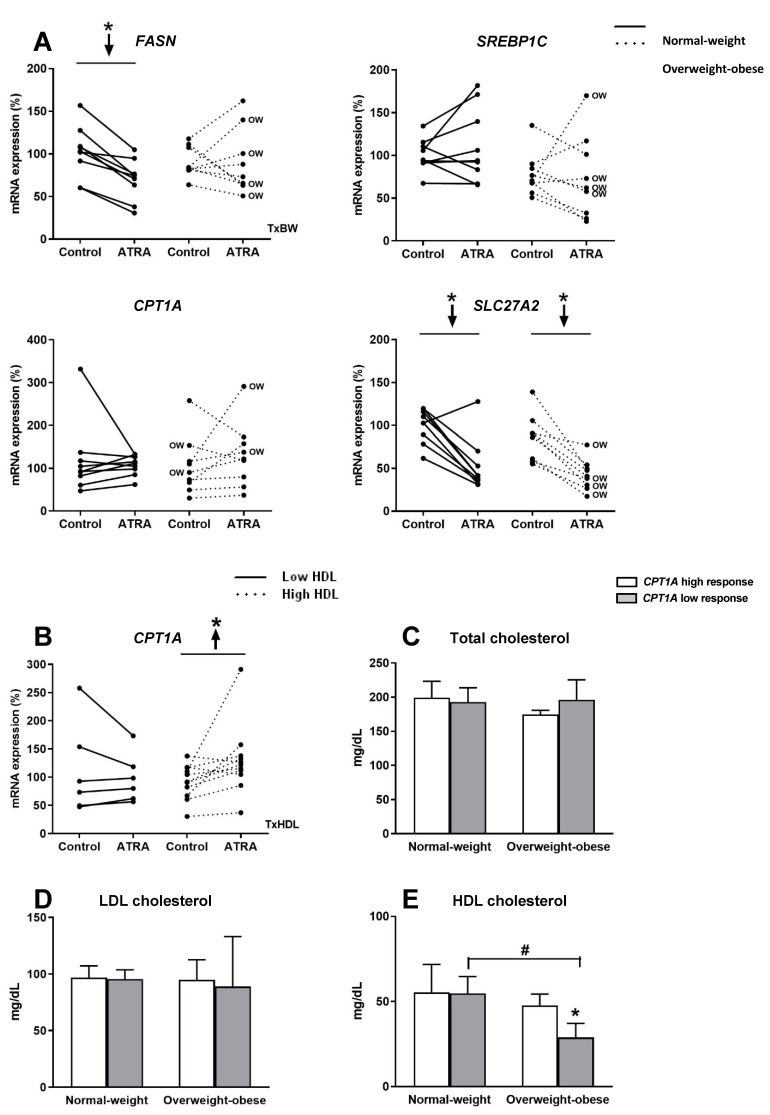
(**A**) Effect of ATRA treatment on mRNA expression of key fatty acid synthesis genes (*FASN* and *SREBP1C*), the key fatty acid oxidation gene (*CPT1A*), and a fatty acid transport gene (*SLC27A2*), in isolated PBMC from NW and OW-OB individuals. * *p* < 0.05 was calculated using a paired *t*-test. Two-way repeated measures ANOVA was performed to compare the different effect of treatments and body weight on gene expression. T × BW, interactive effect between treatment and body weight of PBMC donors; if not indicated no interactive effect was found (detailed results are shown in Table 2A). OW indicates the overweight subjects within individuals of the overweight-obese group. Down arrows represent decreased expression. (**B**) Effect of ATRA treatment on mRNA expression of *CPT1A* in isolated PBMCs from individuals with low and high HDL-cholesterol levels. One outlier data was discarded in the high HDL group and, thus, results from one volunteer were not considered. * *p* < 0.05 was calculated using a paired *t*-test. Two-way repeated measures ANOVA was performed to compare the different effect of treatments and HDL levels. T × HDL, interactive effect between treatment and HDL levels of PBMC donors (detailed results are shown in Table 2C). (**C**) Comparison of plasma total cholesterol, (**D**) LDL-cholesterol, and (**E**) HDL-cholesterol levels in NW and OW-OB individuals with low and high *CPT1A* response to ATRA in PBMCs, taking as reference value the medium response of all the subjects. Mean ± SD is represented. *, differences between low and high *CPT1A* response within the same body weight group (*p* < 0.05, paired *t*-test); #, differences between NW and OW-OB individuals within the same category of gene expression (*p* < 0.05, unpaired *t*-test). No interactive effect was found (detailed results are shown in Table 2D).

The mRNA levels of the key lipogenic gene *FASN*, decreased in PBMCs of the NW group after incubation with ATRA; this inhibitory effect was not observed in the OW-OB group. However, ATRA treatment did not affect *SREBP1C* expression, which codes for a key lipogenic transcription factor (Figure 5A). We also analyzed the regulation of *SLC27A2*, whose coded protein is involved in fatty acid transport, which facilitates the uptake of long chain fatty acids [36,37]. Interestingly, *SLC27A2* expression in PBMCs decreased in both groups of participants in response to ATRA treatment (Figure 5A). Regarding the key gene involved in fatty acid beta-oxidation, *CPT1A*, no effect was observed in PBMCs after ATRA administration either in NW or OW-OB group (Figure 5A). Considering the previously described role of *CPT1A* as a marker of the metabolic status both in animals and humans [7,26], we divided subjects considering anthropometric and biochemical parameters related to metabolic risk in order to analyze possible associations between these parameters and *CPT1A* response. Taking into account fat mass content, circulating triglycerides, total and LDL-cholesterol, glucose and insulin levels, and HOMA-IR index no differences were detected in terms of *CPT1A* expression response (data not shown). Interestingly, when we divided the overall group of subjects by their plasma HDL levels (low vs. high), we found that subjects with high HDL levels were those whose PBMCs showed an increased expression of *CPT1A* after incubation with ATRA (Figure 5B). On the other hand, subjects with lower levels of HDL did not display this response (Figure 5B). To further examine the potential interaction between *CPT1A* response to ATRA in PBMCs and cholesterol levels of volunteers, NW and OW-OB groups were subdivided into categories according to the *CPT1A* gene expression response (high response vs. low response, taking as reference the median response of all the individuals) (Figure 5C–E). When subjects were subdivided on the basis of *CPT1A* response, NW group did not exhibit differences in HDL levels between high- and low-responder subjects (Figure 5E). However, OW-OB subjects with lower *CPT1A* gene expression response to ATRA exhibited lower levels of HDL-cholesterol compared both with their partners with higher *CPT1A* response and with NW group (Figure 5E). In line with these observations, we found a positive correlation between the % *CPT1A* response and circulating HDL-cholesterol (r = 0.656, *p* = 0.055) in PBMCs from all OW-OB donors, confirming a lower response to ATRA treatment in those OW-OB subjects with lower HDL levels. No correlation between *CPT1A* response and HDL levels was evident when considering data from the NW group (r = −0.004, *p* = 0.992). Likewise, no associations were found regarding total and LDL-cholesterol levels in any of the subgroups in relation to their *CPT1A* response to ATRA (Figure 5C,D). Finally, these correlations observed for *CPT1A* mRNA expression and HDL-cholesterol levels were not observed for any of the other genes analyzed (data not shown), reinforcing the specific interest of this gene as a biomarker in studies of nutrition and obesity.

## 4. Discussion

There is a wide amount of information showing that PBMCs offer an accessible source of transcriptomic-based biomarkers of nutritional and metabolic status, giving the possibility to show the metabolic scenario that occur in other tissues, such as liver, adipose tissue or skeletal muscle [4,38]. Moreover, ex vivo assays of human PBMCs offer a plausible model to test the efficacy and safety of food bioactive compounds [4,11]. Food bioactives are recently receiving an increasing attention in terms of their possible beneficial effects on ameliorating metabolic and physiological alterations related to obesity and its co-morbidities. In the present study, we show that the administration of ATRA regulates the expression of key inflammatory and lipid metabolism genes in isolated human PBMCs maintained ex vivo. These gene expression changes may be modulated in part by nuclear receptors. In fact, *RARα* and *RXRα* were expressed in PBMCs of the participants as stated previously by Szabova et al. [24]. Moreover, we describe that ATRA response in PBMCs is impaired depending on body weight of the donors as well as depending on metabolic risk factors, particularly, HDL-cholesterol levels.

The effects of retinoic acid treatment in animals are well studied. It is known that this retinoid is able to ameliorate the negative effects associated with obesity, improving different processes, such as glucose tolerance, insulin sensitivity, and serum lipid profile [13,14,15]. In fact, a previous study from our group using mice showed that whole blood (that includes PBMCs) reflected gene expression patterns after ATRA treatment that occur in other tissues like liver or adipose tissue [39]. However, in humans, the physiological and metabolic effects of retinoic acid are controversial [20]. First of all, ATRA is a minor constituent of the diet and represents less than 5% of total vitamin A in the tissues of healthy animals and humans [40]. In fact, unlike vitamin A or carotenoids, ATRA is not available as a dietary supplement. Therefore, exposure to ATRA is limited, for all practical purposes, to the oral or topical treatment of medical disorders. The use of ATRA or its active derivatives at pharmacological doses is associated to a wide range of side-effects, such as disturbed glucose metabolism, impairment of insulin sensitivity, and increase in serum levels of total cholesterol and triglycerides and decrease in serum levels of HDL-cholesterol [41]. Therefore, the subjects receiving treatment with retinoids could be at greater risk of developing metabolic syndrome in later life. However, numerous studies in humans have described an inadequate vitamin A status in overweight and obese population [42,43]. Despite the fact that the concentration of ATRA in human plasma represents only a small part of all-trans-retinol (<1%) and that it is rapidly metabolized in the body [40], Liu et al. [23] described that serum levels of ATRA in humans are inversely correlated with the risk of metabolic syndrome, regardless of adiposity and insulin resistance. Hence, probably the doses and the routes through which vitamin A derivatives are taken are particularly relevant, as it seems there is a delicate balance between promoting negative effects on lipid profile and ameliorating lipid metabolism.

To the best of our knowledge, although there are few studies analyzing the effects of RA or its derivatives on energy metabolism and inflammatory response in animal and human cell lines [12,35,44,45], there are no studies analyzing the expression of lipid metabolism-related genes in PBMCs after ATRA treatment. Moreover, none of either in vitro or in vivo studies using pharmacological doses of RA or of its derivatives directly compare the differential effects of retinoic acid treatment taking into account metabolic syndrome-related features.

Regarding the effects of ATRA administration on inflammation, our results point towards an anti-inflammatory profile after ATRA administration as has been stated in different cell lines [45,46] and in animal models with inflammatory-related diseases [47,48]. We found a significant reduction in the expression of key inflammatory genes (*NFKB*, *TNFα,* and *TLR2*) in PBMCs from both NW and OW-OB subjects after ex vivo ATRA treatment. Moreover, even if *MCP1* (a chemotactic protein) expression was increased in the two studied groups and *IL6* expression was increased in NW subjects, the released IL6 and TNFα levels in the culture media were lower in PBMCs from NW donors, as well as TNFα levels in PBMCs from OW-OB donors.

Although the analysis of inflammatory genes did not show relevant differences between NW and OW-OB subjects, it is well known that obesity is related to an impaired nutritional response at gene expression level in key metabolic tissues, which is reflected in PBMCs [5,31]. Moreover, we previously found using the same ex vivo system of human PBMCs that subjects with higher BMI showed up an impaired response either of key lipid metabolism and inflammatory genes to long chain n-3 polyunsaturated fatty acids [11]. In fact, when the group of subjects was divided by their BMI, we found that there was a reduction in mRNA levels of both retinoid nuclear receptors in PBMCs of the NW group (*RARα* and *RXRα*). Nevertheless, this auto-regulatory mechanism of the retinoid effects was impaired in the OW-OB group. Probably, these nuclear receptors are mediating the downstream effects of ATRA on the expression of energy metabolism-related genes [18]. The same lack of response was seen in the case of *FASN* gene expression, a key lipogenic gene, whose expression decreased in the NW group but not in the OW-OB group. However, we did not find changes in *CPT1A* in response to ATRA treatment in any of the two studied groups. *CPT1A* codes for a rate-limiting enzyme of fatty acid oxidation, and it is described in animal tissues [13,14] and cell lines [49] that its gene expression is enhanced by ATRA treatment.

In spite of the fact that BMI is the most used measure to classify body weight and obesity, it is well known that it is not the best predictor of metabolic health [50]. Thus, we divided the gene expression response to ATRA taking into account other metabolic syndrome-related features collected from the participants. By doing this, we found an interesting association between HDL levels and *CPT1A* response to ATRA, an association that was not observed either for total or LDL-cholesterol levels, and was exclusive for *CPT1A*, not being observed either for the rest of the genes analyzed. We have shown that individuals with higher levels of HDL display the expected enhanced *CPT1A* expression in PBMCs after ATRA administration. Nevertheless, this response is missing in subjects with levels of HDL below 40 mg/dL, which has been associated to an increased risk of coronary risk [51]. An increasing number of studies show the importance of HDL levels by themselves as a risk factor of cardiovascular disease and HDL is considered as a better risk predictor than LDL [52,53,54]. Therefore, our data reinforce the importance of HDL as predictor of metabolic disturbances, in this case, studied as a lack of *CPT1A* response in PBMC to ATRA treatment. Moreover, our data suggest an association may exist between BMI, HDL levels, and *CPT1A* response to ATRA in PBMCs. That is, here we show that *CPT1A* expression after ATRA treatment is independent of HDL levels in PBMCs from NW subjects but not in the OW-OB group. Remarkably, the OW-OB participants with lower levels of HDL-cholesterol showed an attenuate response to ATRA regarding *CPT1A* expression. This regulation/impaired regulation of *CPT1A* response to ATRA, more related to HDL than to BMI is of interest, as it could be used as a new marker to diagnose metabolic risk with independence of the presence of overweight or obesity, as it is well known that also normal-weight individuals can have increased metabolic syndrome risk [55]. In the same line, previous data from our group described the utility of the analysis of impaired *CPT1A* response to fasting as a marker of metabolic risk (liver steatosis and insulin resistance) in metabolically obese but normal-weight rodents [26]. Moreover, we also showed, in humans, an association between the expression levels of *CPT1A* in whole blood and metabolic alterations associated with childhood obesity [7].

Concerning potential limitations of the study performed, they could be more related to the PBMC ex vivo system per se. PBMCs express a large amount of human genome, but some specific genes of interest may not be expressed or expressed in low amounts in these cells. Thus, even though we demonstrated the usefulness of this system to study the impact of ATRA on key lipid metabolism and inflammatory genes, it could not work so well to study some other specific pathways. Although we obtained clear results and the sample size is appropriate for a controlled ex vivo study, interindividual variability exists, and the use of a higher number of individuals could have helped to get additional statistical differences in our study. Finally, our study was limited to men, so a careful consideration about gender differences should be considered in future studies, including women, as well as individuals from different ethnicities, in order to better represent the whole population.

## 5. Conclusions

An ex vivo system of human PBMCs (derived from men) is able to respond to food bioactive compounds, in this case to ATRA administration, providing a useful, secure, and fast tool to go in depth on the effects of vitamin A derivatives on health. Moreover, PBMC response to ATRA can be influenced/impaired by the presence of overweigh-obesity but also by other metabolic syndrome features, such as low circulating HDL levels. Particularly, *CPT1A* response to ATRA is impaired in those individuals with higher metabolic risk (overweight-obesity and low HDL levels), providing additional evidences of its previously reported role as a metabolic risk predictive biomarker. Finally, our data point to plasmatic levels of HDL-cholesterol to be further explored as a parameter to take into account per se, independently of BMI, which can affect the response to nutritional factors and/or dietary interventions on humans. 

## Figures and Tables

**Table 1:** Nucleotide sequences of primers and amplicon size used for real-time RT-qPCR amplification in human peripheral blood mononuclear cells (PBMCs).

Gene	Forward Primer (5′-3′)	Reverse Primer (3′-5′)	Amplicon Size (bp)
Lipid metabolism genes			
*FASN*	GAGGAAGGAGGGTGTGTTTG	CGGGGATAGAGGTGCTGA	160
*SREBP1C*	TGAGGACAGCAAGGCAAAG	CAGGACAGGCAGAGGAAGAC	108
*CPT1A*	GATTTTGCTGTCGGTCTTGG	CTCTTGCTGCCTGAATGTGA	192
*SLC27A2*	TTTCAGCCAGCCAGTTTTG	TCTCCTCGTAAGCCATTTCC	157
Inflammatory genes			
*CRP*	TCGTATGCCACCAAGAGACA	CCCATCTACCCAGAACTCCA	183
*IL6*	ATGTGTGAAAGCAGCAAAGAG	CACCAGGCAAGTCTCCTCAT	111
*MCP1*	ATCAATGCCCCAGTCACCT	TCCTGAACCCACTTCTGCTT	173
*NFKB*	AGCAATCATCCACCTTCATTC	AGCAAATCCTCCACCACATC	159
*TLR2*	GATGCCTACTGGGTGGAGAA	AAAAGACGGAAATGGGAGAAG	224
*TNFα*	TGGGCAGGTCTACTTTGGGAT	AGAGGTTGAGGGTGTCTGAA	117
Retinoid receptors			
*RARα*	GCTTCACCACCCTCACCAT	GTCTCCGCATCATCCATCTC	235
*RXRα*	ACGAGAATGAGGTGGAGTCG	ATGTTGGTGACAGGGTCGTT	157
Reference gene			
*RPLP0*	ACAACCCAGCTCTGGAGAAA	TGCCCCTGGAGATTTTAGTG	240

For lipid metabolism genes: *FASN*, fatty acid synthase; *SREBP1C*, sterol regulatory element-binding protein 1c; *CPT1A*, carnitine palmitoyl-transferase 1α and *SLC27A2*, solute carrier family 27; for inflammatory genes: *CRP*, C-reactive protein; *IL6*, interleukin 6; *MCP1*, monocyte chemoattractant protein 1; *NFKB*, nuclear factor kappa B; *TLR2*, toll-like receptor 2 and *TNFα*, tumour necrosis factor α; for retinoid receptors: *RARα*, retinoic acid receptor α and *RXRα* and retinoid X receptor α; and reference gene: *RPLP0*, ribosomal protein, large, P0.

**Table 2:** *p*-Values and effect size for the interactive effects analyzed by two-way ANOVA.

(A)	Gene Expression (%)	Treatment × Body Weight (T × BW)	Interactive Effect Size
	*RXRα*	*p* = 0.075	Ƞp^2^ = 0.184
	*RARα*	***p* = 0.037**	Ƞp^2^ = 0.246
	*CRP*	*p* = 0.603	Ƞp^2^ = 0.017
	*IL6*	*p* = 0.471	Ƞp^2^ = 0.033
	*MCP1*	*p* = 0.689	Ƞp^2^ = 0.010
	*NfKβ*	*p* = 0.245	Ƞp^2^ = 0.083
	*TLR2*	*p* = 0.937	Ƞp^2^ = 0.000
	*TNFα*	***p* = 0.041**	Ƞp^2^ = 0.237
	*FASN*	***p* = 0.027**	Ƞp^2^ = 0.272
	*SREBP1C*	*p* = 0.853	Ƞp^2^ = 0.002
	*CPT1A*	*p* = 0.322	Ƞp^2^ = 0.061
	*SLC27A2*	*p* = 0.768	Ƞp^2^ = 0.006
**(B)**	**Cytokine levels (pg/mL)**	**Treatment × body weight (T × BW)**	**Interactive effect size**
	IL6	*p* = 0.641	Ƞp^2^ = 0.016
	TNFα	*p* = 0.227	Ƞp^2^ = 0.096
**(C)**	**Gene expression (%)**	**Treatment × HDL levels (T × HDL)**	**Interactive effect size**
	*CPT1A*	*p* = 0.060	Ƞp^2^ = 0.216
**(D)**	**Parameters (mg/dL)**	**Body weight × CPT1A response (BW × CPT1A)**	**Interactive effect size**
	Total-cholesterol	*p* = 0.244	Ƞp^2^ = 0.111
	LDL-cholesterol	*p* = 0.835	Ƞp^2^ = 0.003
	HDL-cholesterol	*p* = 0.090	Ƞp^2^ = 0.191

Results obtained in the different ANOVA analysis shown in Figure 2, Figure 3, Figure 4 and Figure 5. (**A**)–(**C**): two-way repeated measures ANOVA. (**D**): two-way ANOVA. *p*-values and the interactive effect size (Ƞp^2^ = partial eta squared) are shown. Effects with a *p* < 0.05 are bolded.
